# Impact of the Allergic Asthma on Foot Health-Related Quality of Life and Depression: A Novel Case-Control Research

**DOI:** 10.3390/medicina55050124

**Published:** 2019-05-08

**Authors:** Daniel López-López, Roi Painceira-Villar, Vanesa García-Paz, Ricardo Becerro-de-Bengoa-Vallejo, Marta Elena Losa-Iglesias, David Rodríguez-Sanz, César Calvo-Lobo

**Affiliations:** 1 Research, Health and Podiatry Unit, Department of Health Sciences, Faculty of Nursing and Podiatry, Universidade da Coruña, Ferrol 15403, Spain; roi.painceira.villar@udc.es (D.L.-L.); daniel.lopez.lopez@udc.es (R.P.-V.); 2 Departament of Allergology, Complexo Hospitalario Universitario de Ferrol, Ferrol 15405, Spain; vanesa.garcia.paz@sergas.es; 3 Facultad de Enfermería, Fisioterapia y Podología, Universidad Complutense de Madrid, Madrid 28040, Spain; ribebeva@ucm.es; 4 Faculty of Health Sciences, Universidad Rey Juan Carlos, Alcorcón 28922, Spain; marta.losa@urjc.es; 5 Nursing and Physical Therapy Department, Faculty of Health Sciences, Universidad de León, Ponferrada 24401, Spain; ccall@unileon.es

**Keywords:** asthma, depression, foot deformities, foot diseases, quality of life

## Abstract

*Background:* Asthma may be considered as a non-communicable condition associated with higher bronchial responsiveness that may impair quality of life (QoL). *Purpose:* The research aim was to compare scores of depression, as well as general and foot health-related QoL, in patients who suffered from asthma with respect to healthy subjects. *Methods:* A total sample of 152 subjects, median age of 37.00 ± 16.00 years, were recruited from a respiratory and allergy department of a hospital and divided into patients with asthma (*n* = 76) and healthy subjects (*n* = 76). The scores of the Spanish foot health status questionnaire (SFHSQ) domains as well as the Spanish Beck’s Depression Inventory (BDI) scores and categories were collected. *Results:* The only statistically significant difference (*p* < 0.05) was shown for the difference of the FHSQ footwear domain establishing that patients who suffered from asthma presented a worse QoL related to foot health for footwear (lower FHSQ scores) compared to healthy matched-paired participants (higher FHSQ scores). Regarding the rest of the outcome measurements, there were no statistically significant differences (*p* > 0.05) for the other FHSQ domains scores as well as the BDI scores and categories. *Conclusions:* Patients with allergic asthma presented impairment of the QoL related to foot health for footwear, which seemed to be linked to the presence of asthma.

## 1. Introduction

Asthma may be considered as a non-communicable condition associated with higher bronchial responsiveness, various degrees of airflow obstruction and airway bronchial inflammation [1]. Worldwide, asthma may be observed in the general population with a high prevalence (10–25%) [2] and can be related to other health issues, such as anxiety [3], depression [4], cardiovascular problems [5], obesity [6], pain [7], musculoskeletal alterations [7], that may cause daily limitations, work inability and quality of life (QoL) impairments. 

Thus, this problem was avowed by the physicians and government policy-makers as a global priority with worldwide consequences due to its relevant impact on the patients who suffer from this disease [2].

Despite this, the severity and prevalence of the musculoskeletal alterations in patients that present asthmatic symptoms have been poorly evaluated and there is a lack of studies about depression and QoL related to foot health in patients with asthma problems.

Several musculoskeletal alterations may affect the foot, showing a high prevalence of foot deformities and alterations (71–93%), which show a multifactorial origin and may impair the level of dependence, QoL and wellness [8,9,10,11,12]. Furthermore, a recent investigation lead by López-López et al. on 1647 participants showed that 68.7% of these participants presented some type of foot problems, such as hallux valgus abductus, flat foot, metatarsalgia, keratosis, heel pain, onychocryptosis, toe deformities, pes cavus, and Morton’s Neuroma, which increased with age and were more prevalent in women [8].

According to these previous statements and considering the importance of the control of the foot care in patients with asthma, medical doctors play a key role in diagnosis and treatment of the underlying illness, prevention of further alterations or deformities, and education of the patient in the process related to this illness to find a QoL improvement and wellness for the population.

Thus, the research aim was to compare scores of depression as well as general and foot health-related QoL in patients who suffered from asthma with respect to healthy subjects.

## 2. Methods

### 2.1. Design and Sample

We conducted a descriptive observational investigation according to the Strengthening the Reporting of Observational Studies in Epidemiology Statement (STROBE) guidelines [13]. All patients attended the respiratory and allergy area of the hospital health center called Complexo Hospitalario Universitario de Ferrol, in the town of Ferrol, located at the northwestward of Spain.

Eligible participants comprised patients with asthma (*n* = 76) and healthy subjects (*n* = 76) using a consecutive and non-randomized sampling technique for all participants who were enrolled in this study and recruited from the hospital. The criteria for participation and inclusion were: (1) age higher than eighteen years old, (2) patients with allergic asthma (case group) and healthy subjects without asthma and medical problems (control group), and (3) signature of their informed consent forms. The criteria for exclusion of participants were: (1) smokers and ex-smokers, (2) history of trauma or surgery related to foot and limb, (3) a history of previous comorbidities, (4) utilization of antiallergic immunotherapy, (5) no consent in written form and (6) inability to understand the rules of participating in this investigation.

### 2.2. Procedure

Baseline measurements comprised general questions related to (1) general health, (2) socio-demographic variables (gender, age, smoking habit, and work status), (3) details about comorbid conditions (anxiety, depression, diabetes, obesity, musculoskeletal problems, and vascular alterations), and (4) usual sports activities. Furthermore, specific health questions related to asthma, such as (5) duration of the disease, (6) symptoms of the disease in the last month and (7) current medications, were recorded. 

Afterwards, a senior allergologist physician assessed and analyzed airways alterations by means of a Datospir 600 spirometer (SILBELMED; Barcelona, Spain), which is a validated device for this purpose [14,15]. This protocol followed the international guidelines registering scores of peak expiratory volume in one second (FEV1) for diagnosis of the asthma condition with twelve percent and two hundred milliliters higher in FEV1 [16].

After that, a senior podiatrist researcher completed an overall physical exam for every patient who recorded anthropometrics scores such as: (1) height, (2) weight and (3) body mass index calculation of each patient. 

Next, participants completed the Spanish foot health status questionnaire (SFHSQ) [17]. This survey was translated to Spanish and has been demonstrated to be a validated tool used for evaluations of the overall and specific foot health related to QoL, which is composed of three main sections [18].

The first section of the SFHSQ included 13 questions associated with foot health: (1) foot function, (2) foot pain, (3) footwear, and (4) general foot health. Also, this initial section has demonstrated a great grade of essence, criterion and construct validity with an alpha of Cronbach between 0.89–0.95, retest reliability with an intraclass correlation coefficient between 0.74 and 0.92 [19], and has shown to be a proper tool to evaluate the foot health-related QoL in several conditions such as: foot problems [8], foot pain [20], breast cancer [21], university students [12], Alzheimer disease [9] diabetes [22], among other general diseases and specific alterations.

The second section comprised questions adapted from the short form—36 questions associated with overall health including domains, such as: (1) general health, (2) physical activity, (3) social capacity and (4) vigor [23]. 

The third section showed findings of socio-demographic variables and comorbid conditions. 

The survey did not provide a full overall score but rather it generated scores for the eight domains. All items were evaluated using a software (FHSQ 1.03, Care Quest, Brisbane, Australia) and all scores varied from 0 (poor health) to 100 (optimal health).

Lastly, all participants also responded to the Spanish Beck Depression Inventory (SBDI) [24]. This survey was translated to Spanish and has demonstrated to be a validated tool used for evaluation of depression which was composed of 21 questions and each item was scored on a sub-scale between zero and three points, providing a global score between 0 and 63 points [25]. The analyses of the scores were performed according to the following categories: (1) from zero to nine as without depression, (2) from 10 to 15 as mild depression, (3) from 16 to 23 as moderate depression, (4) from 24 to 57 as severe depression. This tool may be considered as an easy and precise test for the assessment of subjects with signs of depression [26].

### 2.3. Ethics Considerations

The present project of research got a favorable report issued by the local Ethics and Investigation Committee of the Universidade da Coruña with record number CEID-UDC 2018-0022 in the town of A Coruña (Spain), and all participants signed their consent informs in written form, previously to their inclusion in this investigation. The ethical standards for human research and the Declaration of Helsinki (World Medical Association) and rules from other appropriate national/institutional organizations were respected.

### 2.4. Sample size Calculation

The calculation of the sample size was carried out through the between-two-groups differences of independent samples using the G*Power software (version 3.1.9.2, Universität Düsseldorf, Düsseldorf, Germany) and based on the scores of the general foot health domain of the FHSQ from a pilot study (*n* = 20) with two groups (mean ± SD), 10 patients diagnosed with asthma (for case group, 53.00 ± 27.38 points) and 10 healthy matched-paired subjects (for control group, 64.75 ± 30.72 points). Furthermore, one-tailed hypothesis, the effect size of 0.40, probability of α-error of 0.05, power (probability of 1-β error) of 0.80 and allocation ratio (N2/N1) of one were used for the calculation of the sample size. Thus, the total sample size of 152 participants, 76 patients diagnosed with asthma and 76 healthy matched-paired participants, was determined. 

### 2.5. Statistical Analyses

Statistical analysis was performed through the 24.0v SPSS software (IBM Corp., Armonk, NY, USA) considering an alpha error of 0.05 for a 95% confidence interval (CI). 

Considering quantitative data, the test of Kolmogorov-Smirnov was applied to assess normality. All data were distributed as non-parametric data (Kolmogorov–Smirnov test showed a *p*-value lower than 0.05) and were detailed as the median ± interquartile range (IR) and range (minimum–maximum), and differences between both groups were compared by the Mann–Whitney *U* tests of independent samples.

Regarding categorical data, frequencies and percentages were used to detail these values, and differences between both groups were compared by the Fisher exact test (sex variable) or the Chi squared test (BDI category). 

## 3. Results

### 3.1. Descriptive Data

A sample of 152 participants completed the study and was divided into patients diagnosed with asthma (for case group, *n* = 76) and healthy matched-paired subjects (for the control group. *n* = 76) showing an age distribution from 18 to 65 years old. The sample included 58 (38.1%) males and 94 (61.9%) females. Statistically significant differences were not shown (*p* > 0.05) between both groups for descriptive data (Table 1). 

### 3.2. Outcome Measurements

**Figure 1:**
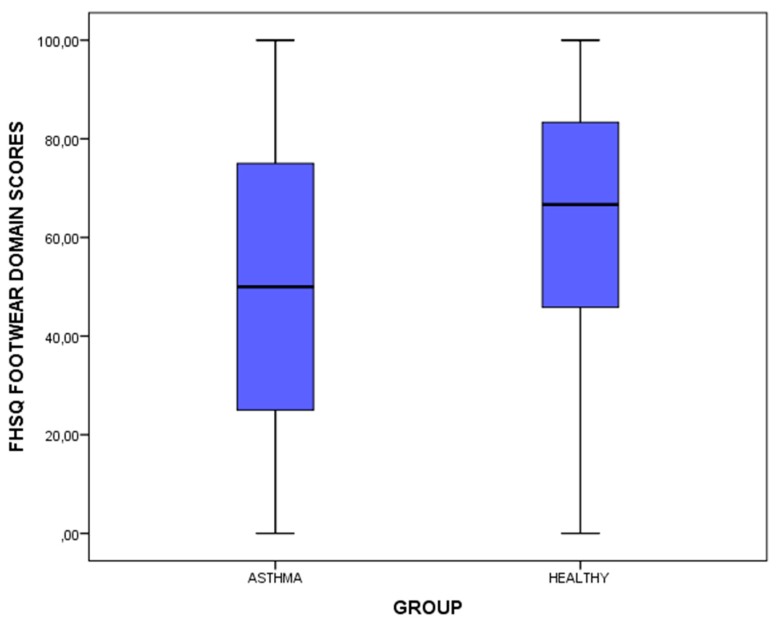
Box-plot to illustrate the differences of the FHSQ footwear domain between the patients diagnosed with asthma and healthy matched-paired controls- *Abbreviations*: FHSQ, Foot Health Status Questionnaire.

The only statistically significant difference (*p* < 0.05) was shown for the difference of the FHSQ footwear domain establishing that patients who suffered from asthma presented a worse QoL related to foot health for footwear (lower FHSQ scores) compared to healthy matched-paired participants (higher FHSQ scores). Regarding the rest of the outcome measurements, there were no statistically significant differences (*p* > 0.05) for the other FHSQ domains scores as well as the BDI scores and categories (Table 2 and Figure 1). 

## 4. Discussion

The goal of this investigation was to compare scores of depression as well as general and foot health-related QoL in patients who suffered from asthma with respect to healthy subjects.

Thus, foot care may be considered as a key objective in patients with allergic asthma due to the increased epidemiology prevalence of musculoskeletal pain in various anatomical areas with a great health impact in patients who suffer from this disease, which may be recognized as a global problem for the public health system according to Barrick et al., who evaluated a sample of 91,642 children with allergic diseases associated with bone problems and severe asthma [27]. In addition, various researchers have concluded the presence of impairment on the QoL in people who suffered from asthma [28,29,30]. 

Nevertheless, there is a lack of investigations of this problem and the impact of the QoL linked to foot health. Thus, the findings of our research confirm that patients with allergic asthma presented impairment on the QoL related to foot health for footwear, compared to healthy subjects without allergic asthma with normalized reference scores.

Considering these findings, the study of the magnitude of the health impairment seems to be important. It stresses the necessity of the assessment of the feet, which should be carried out by the physician as well as the podiatric health care, to prevent the appearance of diseases and conditions of the feet. In line with prior studies in patients with foot problems [8], foot pain [20], breast cancer [21], Alzheimer disease [9] or diabetes [22], the present study also determined that footwear could play a key role in the QoL related to foot health in asthmatic patients. This fact may be a key point that would enable the improvement of the health, QoL and wellness in patients with allergic asthma. According to our findings of the foot health-related QoL impairment in these patients, future interventional studies should evaluate the effects of wearing different footwear on the QoL related to foot health in patients who suffer from asthma.

The comparison of the effect of our findings on other investigations was not possible due to variations in methodological and criteria differences, moreover, we have not been capable to find any paper linking QoL to foot health in patients with allergic asthma in the bibliography. 

In addition to this, the current study had some limitations. Participants with different characteristics, patients living in different locations and greater sample size should be considered to enhance the strength of our research and help to recognize the presence of these problems in different populations who suffer from asthma as a mechanism involved in the QoL impairment related to foot health.

## 5. Conclusions

These novel findings show that patients with allergic asthma presented an impairment on the QoL related to foot health for footwear, which seemed to be linked to the presence of asthma. Therefore, correct care and evaluation of the overall foot health may be a key focus to avoid the development of alterations, soreness, infections or disorders along the control process of the asthmatic population health.

## Figures and Tables

**Table 1:** Descriptive data of the patients diagnosed with asthma and healthy matched-paired controls.

Descriptive Data		Total Group (*n* = 152)	Asthma (*n* = 76)	Healthy (*n* = 76)	*p*-Value
Age (years)		37.00 ± 16.00 (18–65)	36.00 ± 16.50 (20–65)	38.00 ± 16.75 (18–65)	0.368^†^
Weight (kg)		69.00 ± 20.65 (44–120)	69.50 ± 22.00 (44–120)	69.00 ± 18.75 (45-106)	0.900^†^
Height (m)		1.65± 0.14 (1.43–1.97)	1.67 ± 0.15 (1.50–1.97)	1.64 ± 0.13 (1.43–1.87)	0.361^†^
BMI (kg/m^2^)		24.44 ± 5.73 (17.21–39.18)	24.06 ± 6.18 (17.21–39.18)	24.57 ± 5.41 (17.30–34.72)	0.531^†^
Sex	Male	58 (38.1%)	31 (40.8%)	27 (35.5%)	0.617^‡^
	Female	94 (61.9%)	45 (59.2%)	49 (64.5%)	

BMI, body mass index. ^†^, median ± interquartile range, range (min–max) and Mann–Whitney *U* test were used. ^‡^, frequency, percentage (%) and Fisher exact test were utilized. In all the analyses, *p* < 0.05 (with a 95% confidence interval) was considered statistically significant.

**Table 2:** Comparisons of FHSQ domains scores and BDI scores and categories between the patients diagnosed with asthma and healthy matched-paired controls.

Outcome Measurements		Total Group (*n* = 152)	Asthma (*n* = 76)	Healthy (*n* = 76)	*p*-Value (Asthma vs. Healthy)
FHSQ foot pain		87.50 ± 15.63 (0–100)	87.50 ± 21.25 (16.88–100)	87.50 ± 17.97 (0–100)	0.485^†^
FHSQ foot function		93.75 ± 18.75 (6.25–100)	93.75 ± 17.19 (25–100)	93.75 ± 18.75 (6.25–100)	0.997^†^
FHSQ footwear		58.33 ± 41.67 (0–100)	50.00 ± 50.00 (0–100)	66.56 ± 39.58 (0–100)	**0.003**^†^
FHSQ general foot health		72.50 ± 35.00 (0–100)	60.00 ± 35.00 (12.5–100)	76.25 ± 42.50 (0–100)	0.107^†^
FHSQ general health		70.00 ± 40.00 (10–100)	70.00 ± 30.00 (20–100)	70.00 ± 40.00 (10–100)	0.383^†^
FHSQ physical activity		94.44 ± 16.67 (22.22–100)	94.44 ± 16.67 (44.44–100)	94.44 ± 16.67 (22.22–100)	0.529^†^
FHSQ social capacity		100.00 ± 25.00 (0–100)	100.00 ± 25.00 (25–100)	100.00 ± 25.00 (0–100)	0.356^†^
FHSQ vigour		56.25 ± 31.25 (12.5–100)	56.25 ± 31.25 (12.5–100)	56.25 ± 31.25 (12.5–100)	0.626^†^
BDI scores		4.00 ± 7.00 (0–25)	4.00 ± 6.00 (0–24)	3.00 ± 7.00 (0–25)	0.439^†^
BDI category*	no depression	124 (81.6%)	62 (81.6%)	62 (81.6%)	0.928^‡^
	Mild	17 (11.2%)	8 (10.5%)	9 (11.8%)	
	Moderate	11 (7.2%)	6 (7.9%)	5 (6.6%)	
	Severe	0 (0%)	0 (0%)	0 (0%)	

BDI, Beck depression inventory, FHSQ, Foot health status questionnaire. ^†^ Median ± interquartile range, range (min–max) and Mann–Whitney *U* test were used. ^‡^ Frequency, percentage (%) and Chi-squared test (χ^2^) were utilized. * BDI domains were divided as: (1) 0 to 9 points without depression, (2) 10 to 15 points: mild depression, (3) 16 to 23 points: moderate depression, (4) 24 to 57 points: severe depression. In all the analyses, *p* < 0.05 (with a 95% confidence interval) was considered statistically significant (**bold**).
